# Older adult maintaining and improving health self-management through peer supported SMART goal setting

**DOI:** 10.1093/geroni/igab046.2331

**Published:** 2021-12-17

**Authors:** Mary Hynes, Nicole Anderson, Monika Kastner, Arlene Astell

**Affiliations:** 1 University of Toronto, Toronto, Ontario, Canada; 2 North York General Hospital, North York, Ontario, Canada

## Abstract

Non-medical interventions to address risk factors (such as reducing smoking, increasing physical activity, and tackling limited social interaction) are needed to help tackle escalating social and financial health costs. Peer supported interventions have been used successfully to support persons’ health self-management; however, there is limited evidence for group interventions facilitated by older adults. A proof-of-concept study by the first author demonstrated the potential of older adults meeting in groups to each create and follow through with a single SMART goal for any area of health over one-month. This study extends SMART goal setting to enhancing health management over six months. Older adult participants from across Ontario will attend virtual SMART goal setting group sessions followed by six monthly support group meetings where they are free to choose any goal, whether a mitigation or a new behavior. Each month the facilitator will assist participants to continue, modify, or set a new goal. At the end participants will complete surveys about their satisfaction with the method, their results and their desire to continue with SMART goals. They will also be asked if they would like to facilitate new groups to continue the spread of peer-supported SMART goal groups. This study is designed to empower older adults to maintain or improve management of their physical, psychological, and/or social health. It will reveal the impact of an older adult created and guided group health intervention on feelings of self-efficacy and well-being.

